# Searching practices and inclusion of unpublished studies in systematic reviews of diagnostic accuracy

**DOI:** 10.1002/jrsm.1389

**Published:** 2020-02-05

**Authors:** Daniël A. Korevaar, Jean‐Paul Salameh, Yasaman Vali, Jérémie F. Cohen, Matthew D. F. McInnes, René Spijker, Patrick M. Bossuyt

**Affiliations:** ^1^ Department of Respiratory Medicine, Amsterdam University Medical Centres University of Amsterdam The Netherlands; ^2^ Clinical Epidemiology Program Ottawa Hospital Research Institute Ottawa Canada; ^3^ Department of Clinical Epidemiology, Biostatistics and Bioinformatics, Amsterdam University Medical Centres University of Amsterdam Amsterdam the Netherlands; ^4^ Department of General Pediatrics and Pediatric Infectious Diseases, Necker‐Enfants Malades Hospital, Assistance Publique‐Hôpitaux de Paris Paris France; ^5^ Inserm UMR 1153 (Centre of Research in Epidemiology and Statistics) Paris Descartes University France; ^6^ Department of Radiology University of Ottawa Ottawa Canada; ^7^ Cochrane Netherlands, Julius Center for Health Sciences and Primary Care, University Medical Centre Utrecht Utrecht University The Netherlands; ^8^ Medical Library, Amsterdam University Medical Centers University of Amsterdam Amsterdam the Netherlands

## Abstract

**Introduction:**

Many diagnostic accuracy studies are never reported in full in a peer‐reviewed journal. Searching for unpublished studies may avoid bias due to selective publication, enrich the power of systematic reviews, and thereby help to reduce research waste. We assessed searching practices among recent systematic reviews of diagnostic accuracy.

**Methods:**

We extracted data from 100 non‐Cochrane systematic reviews of diagnostic accuracy indexed in MEDLINE and published between October 2017 and January 2018 and from all 100 Cochrane systematic reviews of diagnostic accuracy published by December 2018, irrespective of whether meta‐analysis had been performed.

**Results:**

Non‐Cochrane and Cochrane reviews searched a median of 4 (IQR 3‐5) and 6 (IQR 5‐9) databases, respectively; most often MEDLINE/PubMed (n = 100 and n = 100) and EMBASE (n = 81 and n = 100). Additional efforts to identify studies beyond searching bibliographic databases were performed in 76 and 98 reviews, most often through screening reference lists (n = 71 and n = 96), review/guideline articles (n = 18 and n = 52), or citing articles (n = 3 and n = 42). Specific sources of unpublished studies were searched in 22 and 68 reviews, for example, conference proceedings (n = 4 and n = 18), databases only containing conference abstracts (n = 2 and n = 33), or trial registries (n = 12 and n = 39). At least one unpublished study was included in 17 and 23 reviews. Overall, 39 of 2082 studies (1.9%) included in non‐Cochrane reviews were unpublished, and 64 of 2780 studies (2.3%) in Cochrane reviews, most often conference abstracts (97/103).

**Conclusion:**

Searching practices vary considerably across systematic reviews of diagnostic accuracy. Unpublished studies are a minimal fraction of the evidence included in recent reviews.

## 1 INTRODUCTION

Systematic reviews aim to provide a comprehensive and informative summary of the evidence on a certain topic, for example, the effectiveness of an intervention or the accuracy of a diagnostic test.^1^, ^2^ Unfortunately, a reviewer's job is impeded by the fact that approximately half of all initiated biomedical studies are never reported in full in a peer‐reviewed journal.^3^ Unpublished studies are often difficult to identify, making the inclusion of their results in systematic reviews a hazardous task. This may lead to flawed and overoptimistic review conclusions, when studies with more optimistic results are published more often. Among systematic reviews of therapeutic interventions, it has been documented that published trials report, on average, a 9% greater treatment effect than unpublished ones.^4^

For diagnostic accuracy studies, evidence of similar selective publication practices is still scarce, yet increasing. In recent years, a number of evaluations assessed publication rates among completed diagnostic accuracy studies, identifying that approximately a quarter to half of them failed to reach full‐text publication in a peer‐reviewed journal.^5^, ^6^, ^7^, ^8^, ^9^ Two studies evaluated time from study completion to publication among published diagnostic accuracy studies, both concluding that those reporting higher estimates of diagnostic accuracy were published more rapidly.^10^, ^11^ It seems plausible that studies reporting higher estimates of diagnostic accuracy also more often reach publication, although this has yet to be demonstrated.^6^, ^7^, ^8^, ^9^

To prevent the potential bias from relying only on published evidence in systematic reviews, guidance documents invite reviewers to search for studies that are not reported in peer‐reviewed journals but may be identifiable in, for example, proceedings of scientific conferences or prospective trial registries.^12^, ^13^, ^14^, ^15^, ^16^ Making efforts to identify unpublished data may also result in more precise estimates of diagnostic accuracy after meta‐analysis and provide better opportunities to investigate sources of heterogeneity in meta‐regression, which is not always possible in standard systematic reviews of diagnostic accuracy, typically due to small numbers of primary studies.^17^ As such, including unpublished studies may help to reduce avoidable research waste due to a failure to report studies in full.^18^, ^19^

The objective of this study was to assess searching practices among recent systematic reviews of diagnostic accuracy, with a special focus on the identification and inclusion of unpublished studies. We were suspecting that, despite the accumulating evidence that many studies remain unreported, a majority of systematic reviews fails to search for or identify such studies. Given the explicit guidance provided in the *Cochrane Handbook for Diagnostic Test Accuracy Reviews* and the thorough peer‐review process that protocols for Cochrane systematic reviews undergo before they are initiated,^16^ we also evaluated a set of Cochrane reviews.

## 2 METHODS

In this evaluation, unpublished studies were defined as those that had not been reported in full in a peer‐reviewed journal but had only been described or mentioned in, for example, conference abstracts, trial registries, dissertations, repositories, book chapters, or unpublished manuscripts obtained through contact with investigators.

### 2.1 Selection of systematic reviews

Two sets of systematic reviews of diagnostic accuracy were obtained. First, we used a set of systematic reviews identified in a recently published project on reporting quality of systematic reviews of diagnostic accuracy, for which the full search details have been reported elsewhere.^20^ In short, MEDLINE had been searched for systematic reviews of diagnostic accuracy published between 31 October 2017 and 20 January 2018, where the time span had been modulated to reach a convenience sample size of 100 systematic reviews, using the following search strategy: “systematic[sb] AND (sensitivity and specificity[mesh] OR sensitivit*[tw] OR specifit*[tw] OR accur*[tw] OR ROC[tw] OR AUC[tw] OR likelihood[tw]).”

In addition, we obtained a set of Cochrane systematic reviews of similar size by searching the Cochrane Library (www.cochranelibrary.com/cdsr/reviews) filtering the “type” of systematic review by “diagnostic,” without any additional search terms. We searched from inception onwards until we arrived at a convenience sample of 100 Cochrane systematic reviews. The first Cochrane systematic review of diagnostic accuracy was published in October 2009; the 100th in December 2018.

Both non‐Cochrane and Cochrane systematic reviews were included if they had evaluated the diagnostic accuracy of one or more index tests against a reference standard in humans, independent of whether they had been able to include studies or to perform meta‐analysis. Systematic reviews published in languages other than English were excluded.

### 2.2 Data extraction

All data extraction was performed by one investigator (DAK) and all extracted information was checked by a second investigator (JPS or YV), who marked 44 datapoints (out of a total of 10  800) for discussion. Disagreements were resolved through discussion. The complete report of each systematic review was read, and the following characteristics were extracted:

#### 2.2.1 General characteristics of included systematic reviews

We extracted type of systematic review (non‐Cochrane vs Cochrane), first author, number of authors, country of corresponding author, year of publication, type of index test under evaluation (imaging test, laboratory test, another type of test, or multiple types of tests), target condition, language restrictions applied, and whether efforts were made to contact authors of included studies for additional data (eg, in case of incomplete reporting). We also extracted all bibliographic databases searched for the review and whether unpublished studies were explicitly eligible for inclusion.

#### 2.2.2 Additional efforts to identify studies

We extracted whether additional efforts were made to identify potentially eligible (published or unpublished) studies beyond searching bibliographic databases (categorized as screening of reference lists of included studies, screening of review articles or clinical guidelines, screening of articles citing included studies, contacting authors or experts, using a “related articles” search feature, contacting device manufacturers, or other), and whether specific sources of unpublished studies were searched (categorized as sources of conference abstracts, trial registries, or other [including specific sources of theses, dissertations, studies in‐progress or other grey literature]).

#### 2.2.3 Systematic review results

Finally, we also extracted total number of studies included in the systematic review, number of unpublished studies included and through which sources these had been identified, number of identified ongoing unpublished studies (ie, studies that fulfilled the inclusion criteria of the systematic review but had not yet been completed) and their sources, whether at least one meta‐analysis had been performed, whether unpublished studies had been included in a meta‐analysis, and whether the authors had pre‐planned a comparison between published and unpublished studies (or a sensitivity analysis excluding unpublished studies) and what the results of this comparison were.

### 2.3 Data analysis

Quantitative analysis consisted in descriptive statistics. Data on practices for including unpublished studies were reported as frequencies and percentages, or as medians and interquartile ranges (IQR). Data were analyzed for non‐Cochrane and Cochrane systematic reviews separately as we expected considerable differences in searching practices between the two groups, as has been found for systematic reviews of therapeutic studies.^21^ We did not attempt a statistical comparison between non‐Cochrane and Cochrane systematic reviews, as they covered different timeframes; in addition, because we included *all* published Cochrane systematic reviews inference to a larger population does not apply. A comparison between published vs unpublished studies among meta‐analyses containing at least three published and three unpublished studies was pre‐planned but not performed due to limited data, as there were only seven systematic reviews that fulfilled this criterion.

## 3 RESULTS

### 3.1 General characteristics of included systematic reviews

We included 100 non‐Cochrane systematic reviews and 100 Cochrane systematic reviews. An overview of systematic review characteristics and results is provided in Table 1.

**Table 1:** Characteristics of included systematic reviews of diagnostic accuracy

	Non‐Cochrane systematic reviews (n = 100)	Cochrane systematic reviews (n = 100)
**General characteristics and efforts to identify studies**		
Number of authors, median (IQR)	5 (4–7)	7 (6‐8)
*Type of index test*		
Imaging test	60	34
Laboratory test	27	33
Other type of test	9	26
Multiple types of tests	4	7
*Language restrictions*		
No	37	90
Yes	56	6
Not reported	7	4
*Authors contacted for additional data, if needed*		
Yes	31	78
No or not reported	69	22
*At least some requested data obtained after contacting authors*		
Yes	8	52
No or not reported	23	26
*Number of bibliographic databases searched, median (IQR)*	4 (3–5)	6 (5–9)
MEDLINE/PubMed	100	100
EMBASE	81	100
Cochrane Library (including CENTRAL, DARE and/or HTA)	68	71
Web of Science (including CPCI and/or SCI)	42	65
LILACS	13	39
BIOSIS (including BIOSIS Previews and/or BIOSIS Citation Index)	4	36
CINAHL	11	33
PsychINFO	3	27
SCOPUS	21	7
African Index Medicus	2	4
Other	40	85
*Unpublished studies eligible for inclusion*		
(At least one type of) unpublished studies explicitly eligible	10	42
(At least one type of) unpublished studies explicitly not eligible	36	10
Not reported (although some did explicitly search sources of unpublished studies)	54	48
**Additional efforts to identify (published or unpublished) studies**		
*At least one additional effort made*	76	98
Screening of reference lists of included studies	71	96
Searching of relevant review articles or clinical guidelines	18	52
Screening of articles citing included studies	3	42
Contacting authors or experts	6	37
Using a “related articles” search feature	6	32
Contacting device manufacturers	0	9
Other	4	11
**Specific sources of unpublished studies searched**		
*At least one specific source searched*	22	68
Sources of conference abstracts searched	6	45
Conference proceedings of specific conferences	4	18
Databases only containing conference abstracts (ie, CPCI and/or British Library Zetoc conference search)	2	33
Trial registries searched	12	39
ClinicalTrials.gov	7	33
WHO ICTRP	6	32
ISRCTN	1	12
Other	1	3
Other (ie, specific sources of theses, dissertations, studies in‐progress, or other grey literature)	10	15
ProQuest Dissertations and Theses	3	6
OpenGREY	6	4
Other	4	7
**Systematic review results**		
Total number of studies included, median (IQR)	14.5 (10–23)	15.5 (8–41)
At least one unpublished study included in systematic review	17	23
At least one meta‐analysis performed	89	71
At least one unpublished study included in at least one meta‐analysis	14	18
Comparison between published and unpublished studies (or a sensitivity analysis excluding unpublished studies) planned	1	11
Comparison between published and unpublished studies (or a sensitivity analysis excluding unpublished studies) performed	1	2

*Note*: Data are absolute numbers, unless otherwise indicated.

Abbreviation: IQR, inter quartile range.

The median number of authors was 5 (IQR 4‐7) for non‐Cochrane systematic reviews and 7 (IQR 6‐8) for Cochrane systematic reviews. Corresponding authors were mostly from China (n = 28), United States (n = 13) and South Korea (n = 12) for non‐Cochrane systematic reviews, and from the United Kingdom (n = 50), the Netherlands (n = 9) and Australia (n = 8) for Cochrane systematic reviews. The type of index test under investigation was most often an imaging test (n = 60 and n = 34), followed by a laboratory test (n = 27 and n = 33).

Of the non‐Cochrane systematic reviews, 37/100 explicitly reported in their methods section that no language restrictions were applied, compared to 90/100 Cochrane systematic reviews; those that had applied language restrictions usually restricted inclusion to English only (43 of 56, and 4 of 6). Only seven and four systematic reviews did not report whether language restrictions were applied. Efforts to contact authors in case of incomplete or unclear data were announced or reported by 31 non‐Cochrane systematic reviews and by 78 Cochrane systematic reviews. Of these, 13 and 63 reported that the authors of at least one primary study had actually been contacted, whereas the remaining did not report this information. In addition, 8 and 52 reported that at least some requested data had been obtained after contacting authors of primary studies, whereas the remaining 23 and 26 reported that no data had been obtained or did not report this information.

Non‐Cochrane and Cochrane systematic reviews had searched a median of 4 (IQR 3‐5) and 6 (IQR 5‐9) bibliographic databases, respectively. Databases most often searched were MEDLINE/PubMed (n = 100 and n = 100), Embase (n = 81 and n = 100), at least one database within the Cochrane Library (n = 68 and n = 71), and at least one database within Web of Science (n = 42 and n = 65). Regional databases such as Latin American and Caribbean Health Sciences Literature (LILACS) (n = 13 and n = 39) and African Index Medicus (n = 2 and n = 4) were less often searched. This also applied to Chinese databases such as CNKI and WanFang (n = 11 and n = 0 systematic reviews searched at least one Chinese database).

Of the non‐Cochrane systematic reviews, 10 explicitly reported that they considered (at least one type of) unpublished studies for inclusion, or that they had searched for studies independent of publication status/type. In contrast, 36 systematic reviews explicitly reported that (at least one source of) unpublished studies were not eligible for inclusion: 23 referred to conference abstracts and 13 to unpublished, non‐peer reviewed or grey literature studies in general. The remaining 54 non‐Cochrane systematic reviews did not make explicit comments about whether (a type of) unpublished studies were eligible for inclusion, although 13 of these reported having searched in one or more specific sources of unpublished studies, and 10 included at least one unpublished study.

Of the Cochrane systematic reviews, 42 explicitly reported that they considered (at least one type of) unpublished studies for inclusion, or that they searched for studies independent of publication status/type. In contrast, 10 systematic reviews explicitly reported that (at least one source of) unpublished studies were not eligible for inclusion: eight referred to conference abstracts and two to unpublished studies in general. The remaining 48 Cochrane systematic reviews did not make explicit comments about whether (a type of) unpublished studies were eligible for inclusion, although 35 of these reported having searched in one or more specific sources of unpublished studies (eg, conference proceedings or trial registries), and 9 had included one or more unpublished studies.

### 3.2 Additional efforts to identify studies

Additional efforts to identify potentially eligible (published or unpublished) studies beyond searching bibliographic databases were performed by 76 non‐Cochrane systematic reviews and by 98 Cochrane systematic reviews: screening of reference lists of included studies (n = 71 and n = 96), searching of relevant review articles or clinical guidelines (n = 18 and n = 52), screening of articles citing included studies (n = 3 and n = 42), contacting authors or experts (n = 6 and n = 37), using a “related articles” search feature (n = 6 and n = 32), or contacting device manufacturers (n = 0 and n = 9). Other efforts to identify studies included screening reports from World Health Organization (WHO; n = 0 and n = 5), websites such as Food and Drug Administration (FDA; n = 1 and n = 3), or specific journals (n = 3 and n = 2).

Specific sources of unpublished studies were searched by 22 non‐Cochrane systematic reviews, and by 68 Cochrane systematic reviews. These included conference proceedings of specific conferences (n = 4 and n = 18), databases only containing conference abstracts (ie, CPCI and/or British Library Zetoc conference search; n = 2 and n = 33), or trial registries (n = 12 and n = 39), most often ClinicalTrials.gov (n = 7 and n = 33). Other efforts to identify unpublished studies included searching ProQuest Dissertations and Theses (n = 3 and n = 6) and OpenGREY (n = 6 and n = 4).

### 3.3 Systematic review results

The median total number of primary studies included in the systematic reviews was 14.5 (IQR 10‐23) in non‐Cochrane systematic reviews and 15.5 (IQR 8‐41) in Cochrane systematic reviews. At least one unpublished study was included in 17 and 23 systematic reviews; the median number of unpublished studies among these systematic reviews was 1 (IQR 1‐2) and 3 (IQR 1‐3).

In the non‐Cochrane systematic reviews, a total of 2082 primary studies were included. Of these, 39 (1.9%) were unpublished studies; these were conference abstracts (n = 36), a dissertation (n = 1), an unpublished study from the review authors themselves (n = 1), or not reported (n = 1). In the Cochrane systematic reviews, a total of 2780 primary studies were included. Of these, 64 (2.3%) were unpublished studies; these were conference abstracts (n = 61), identified in a trial registry (n = 1), or included in a previous systematic review (n = 2). None of the systematic reviews explicitly reported through which source they had identified the included conference abstracts. Characteristics of the three systematic reviews including the largest numbers of unpublished studies are provided in Table 2.

**Table 2:** Systematic reviews of diagnostic accuracy including the largest numbers of unpublished studies

	Wan and colleagues^22^	Cohen and colleagues^23^	Best and colleagues^24^
Type of systematic review	Non‐Cochrane	Cochrane	Cochrane
Index test	EUS and MRCP	Rapid antigen detection test	Several imaging modalities
Target condition	Idiopathic acute pancreatitis	Group A streptococcus pharyngitis	Focal pancreatic lesions
Number of databases searched	6 ‐PubMed (MEDLINE) ‐EMBASE ‐Cochrane Library (CENTRAL) ‐OVID ‐CNKI ‐Wanfang	6 ‐MEDLINE ‐EMBASE ‐Web of Science (including CPCI and SCI) ‐Cochrane Library (CENTRAL and CDSR) ‐MEDION ‐TRIP	4 ‐MEDLINE ‐EMBASE ‐Web of Science (including SCI) ‐Cochrane Library (CENTRAL)
Conference proceedings of specific conferences searched	Yes “abstracts from recent conferences were searched manually”; no further details	No But CPCI was searched for conference abstracts	No
Trial registries searched	No	No	No
Additional efforts made to identify studies	Yes ‐Review articles	Yes ‐Screening reference lists ‐Screening citing articles ‐Contacting manufacturers ‐Screening review articles ‐Using related articles search feature (in PubMed)	Yes ‐Screening reference lists ‐Screening citing articles ‐Using related articles search feature (in MEDLINE and EMBASE)
Language restrictions	No	No	No
Authors contacted for additional data, if needed	No	Yes “If some data were unclear or missing, we attempted to contact study authors”	Yes “We sought further information from study authors where necessary”
Total number of studies included	34	98	54
Number of unpublished studies Included	12 (35.3%) (all were conference abstracts)	8 (8.2%) (all were conference abstracts)	8 (14.8%) (all were conference abstracts)
Comparison between published and unpublished studies, or sensitivity analysis excluding unpublished studies, planned	Yes “Excluding conference abstracts … showed no influence in the results”	No	Yes Not performed due to “sparseness of the data”

Abbreviations: EUS, endoscopic ultrasound; MCRP, magnetic resonance cholangiopancreatography.

At least one meta‐analysis was performed in 89 non‐Cochrane systematic reviews vs 71 in Cochrane systematic reviews. However, only 14 non‐Cochrane systematic reviews included at least one unpublished study in at least one meta‐analysis vs 18 for the Cochrane systematic reviews. Overall, eight systematic reviews included at least one unpublished study but did not include them in a meta‐analysis; six of these did not perform meta‐analysis at all, and the other two only performed meta‐analysis on a small proportion of included studies providing sufficient data. A comparison between the results of published vs those of unpublished studies (or a sensitivity analysis excluding unpublished studies) was planned according to Section 2 in 1 and 11 systematic reviews. However, only three systematic reviews actually reported such an analysis; one did not observe a significant difference between published and unpublished studies and two identified no influence on the results when excluding unpublished studies. For the remaining nine systematic reviews, the authors indicated that the small number or the absence of unpublished studies withheld them from performing the analysis.

Of the non‐Cochrane systematic reviews, only three explicitly reported whether they had identified ongoing eligible studies (ie, studies that fulfilled the inclusion criteria of the systematic review but had not yet been completed), identifying 0, 2, and 6 ongoing studies. In contrast, 24 Cochrane systematic reviews reported this information: five reported to have identified 0 ongoing studies; the remaining 19 reported to have identified at least one ongoing study (ranging from 1 to 25). Sources through which these 80 ongoing studies were identified were trial registries (n = 56), conference abstracts (n = 5), contact with researchers (n = 2), published in journals (n = 1), and not reported (n = 16).

## 4 DISCUSSION

We observed that efforts to identify eligible studies varied considerably across recently published systematic reviews of diagnostic accuracy. Only a minority of non‐Cochrane systematic reviews reported having searched for studies not reported in journals, and only a small number of systematic reviews had actually included unpublished studies.

This study is not without limitations. Many systematic reviews did not explicitly report whether they had included unpublished studies. We carefully screened the references of studies included in the systematic reviews to check whether these had been published. Although this was done by two authors, we may have missed unpublished studies, which may have led to an underestimation of the number of unpublished studies included in the evaluated systematic reviews.

We acknowledge that our definition of “unpublished studies” may refer to data that is in fact publicly available, for example, reported in conference abstracts or dissertations. Authors of systematic reviews who explicitly reported that unpublished studies were or were not eligible for inclusion may have used a different definition of “unpublished.” This is, for example, illustrated by the fact that we found a systematic review that explicitly excluded “unpublished studies” but had actually included a conference abstract.^25^ Some systematic reviews reported to have obtained additional unreported data by contacting authors of studies published in peer‐reviewed journals. We considered such studies as “published” although the unreported data may have included 2 × 2 tables that ended up in the meta‐analysis.

The adequacy of data extracted in our review completely relies on completeness of reporting in the included systematic reviews. Research has shown that authors of systematic reviews often fail to report critical information.^20^, ^26^, ^27^, ^28^ In such cases, the extracted data may not represent the actual methodology used by the reviewers.

Our search for non‐Cochrane and Cochrane systematic reviews covered different timeframes: October 2017 to January 2018 vs October 2009 to December 2018, respectively. For this reason, we did not perform a formal statistical comparison between the two groups. It seems unlikely that non‐Cochrane systematic reviews published prior to 2017 made more efforts to identify unpublished studies.

The *Cochrane Handbook for Diagnostic Test Accuracy Reviews* explicitly recommends reviewers to locate unpublished studies and to include them in a systematic review to minimize risk of bias.^16^ Our findings show that, even among Cochrane systematic reviews, efforts to identify unpublished studies are often absent or minimal. The fact that only 1.9% of all primary studies included in the non‐Cochrane systematic reviews were unpublished, and only 2.3% of those included in the Cochrane systematic reviews indicate that it is highly likely that such reviews fail to include a considerable amount of completed diagnostic accuracy studies.

This is worrying for multiple reasons. First, despite the fact that time and effort has been put in performing these studies, and patients may have been posed to risk by participating in them, their added value to clinical practice is likely to be nihil. This is a major source of avoidable research waste and can be considered unethical.^18^, ^19^ Identifying and including such studies may lead to more precise meta‐analysis results and provide more room for investigating sources of heterogeneity, thereby increasing research value. Second, publication bias in meta‐analyses lures when the results of unpublished studies are systematically different from those of published studies. Among trials of interventions, for example, it has been shown that those with significant findings are more often published than those without.^29^, ^30^, ^31^ Whether this phenomenon also occurs among diagnostic accuracy studies is largely unclear. Some evidence is hinting towards similar selective reporting practices, although other studies could not confirm this.^6^, ^7^, ^8^, ^9^, ^10^, ^11^ In our study, among the three systematic reviews that made a comparison between published and unpublished diagnostic accuracy studies included in the meta‐analysis (or performed a sensitivity analysis excluding unpublished studies), none found a significant difference.

Systematic reviewers should be aware of other sources of reporting bias as well. Although almost none of the Cochrane systematic reviews applied language restrictions, this was only the case for 37% of non‐Cochrane systematic reviews. This may introduce language bias, where studies in non‐English language produce less optimistic results. Chinese and other regional databases, which have been shown to contain large amounts of studies not available through databases such as Medline and Embase,^32^ were searched in a minority of systematic reviews.

How do systematic reviews of diagnostic accuracy compare to other types of systematic reviews? Several evaluations of search methods among systematic reviews in different fields of research have been performed, with varying results. These also showed that efforts to identify all eligible studies were in many cases suboptimal. Among systematic reviews of adverse effects of medical interventions, for example, 39% searched at least one source of unpublished studies, and 48% of these were able to include unpublished data.^33^ A recent assessment found that 39% of systematic reviews published in 2014 explicitly reported that both published and unpublished studies were eligible for inclusion, whereas 27% explicitly restricted to published studies only, and 34% did not report this information.^21^ Sources of unpublished data, however, were rarely searched; for example, only 19% of systematic reviewers screened trial registries. Another evaluation of grey literature in systematic reviews in child‐relevant Cochrane systematic reviews found that only 5.6% were able to include an unpublished study, and such studies only represented 1.9% of all included studies.^34^

Assessing the risk of publication bias and other reporting biases in a systematic review of diagnostic accuracy is not an easy task. In a set of 114 of such systematic reviews, it was shown that 47 used statistical methods to investigate publication bias.^35^ However, the use of such methods is generally not advised as they may produce inconsistent results (ie, different statistical methods applied on the same dataset may lead to conflicting inference), and because heterogeneity in test accuracy may lead to funnel plot asymmetry not necessarily implying publication bias.^35^, ^36^ For this reason, the Preferred Reporting Items for Systematic Reviews of Diagnostic Test Accuracy (PRISMA‐DTA) guideline does not invite authors to report statistical analyses of publication bias.^26^ Rather than assessing the risk of publication bias statistically, it seems preferred to limit the potential of such bias by making considerable efforts to identify and include unpublished studies.

Previous evaluations have shown that especially conference proceedings and trial registries are excellent sources of unpublished diagnostic accuracy studies. An evaluation of diagnostic accuracy studies registered in ClinicalTrials.gov found that only 54% reached full‐text publication in a peer reviewed journal.^5^ Similar evaluations of publication rates among diagnostic accuracy studies presented at international conferences in the fields of dementia, ophthalmology, radiology, and stroke found that, respectively, 39%, 57%, 71%, and 76% reached full‐text publication.^6^, ^7^, ^8^, ^9^ Unfortunately, our evaluation shows that only a minority of systematic reviews of diagnostic accuracy searched these sources.

Still, identifying unpublished studies may be difficult and time‐consuming, which is illustrated by the fact that even among systematic reviews that made considerable efforts to identify unpublished studies, most only included a small number, if any. Information reported in conference abstracts is often limited, for which reason they may not be picked up by literature searches.^37^, ^38^ In addition, although databases such as Conference Proceedings Citation Index (CPCI), BIOSIS Previews and EMBASE contain large numbers of conference abstracts, many conferences are not covered by these databases. In such cases, proceedings of specific conferences may be difficult to access and, in the absence of an electronic searching feature, may need to be browsed manually. Trial registries such as ClinicalTrials.gov contain large numbers of ongoing and completed diagnostic accuracy studies, but a literature review found that still only 15% of diagnostic accuracy studies published in high‐impact journals were actually registered in a trial registry.^39^ Even when a conference abstract or registered record of an unpublished study is identified, it may be difficult to include the study in a meta‐analysis due to sparse or absent reporting of methodological features or results, prohibiting a proper quality assessment or data extraction.^37^, ^40^ An additional concern is that these unpublished studies usually have not undergone a thorough peer‐review process, and that data may be preliminary.^41^ Future research may focus on establishing the optimal sources of identifying unpublished diagnostic accuracy studies.

Over the past years, registration of clinical trials before inclusion of the first participant in the study has been enforced by numerous organizations, such as the International Committee of Medical Journal Editors (ICMJE).^42^, ^43^ A major advantage of such registration is that all ongoing, completed and terminated trials can be identified and included in literature syntheses. It is highly recommended that researchers also start registering their diagnostic accuracy studies.^44^, ^45^, ^46^ The Standards for Reporting of Diagnostic Accuracy Studies (STARD) group recently established guidance on how to register a diagnostic accuracy study in an informative manner in existing clinical trial registries.^40^, ^47^ It was found that the majority of existing clinical trial registries accept registration of such studies.

In conclusion, although large numbers of diagnostic accuracy studies are never reported in full in a peer‐reviewed journal, they only make up a tiny fraction of the evidence included in systematic reviews. This represents a major source of avoidable waste of research efforts and funds. Failure to include unpublished studies may lead to a partial and biased view of the available evidence. We recommend that reviewers increase their efforts to identify unpublished diagnostic accuracy studies and to include them in their evidence syntheses.

## 5 POTENTIAL IMPACT FOR RSM READERS?

Including unpublished studies in systematic reviews may reduce bias due to selective publication, can increase the power of explorations of heterogeneity in meta‐analysis, and should help in reducing avoidable research waste.

## Supporting information


**Appendix S1:** Supporting Information

## Data Availability

The full data set is available in the Supporting Information S1.
